# Summer Resorts

**Published:** 1859-07

**Authors:** 


### SUMMER RESORTS.

In order to derive the highest advantages as to health, from summer recreations, several considerations ought to be kept in view.

Children who are teething, should be taken without an hour's delay, to the sea shore. The effect is, in multitudes of cases, instantaneous, radical, and almost miraculous. Physicians of observation in large cities will testify, that children in their second summer, in an almost dying condition, begin to improve on their journey to the coast, and within three hours after leaving the heated and sultry atmosphere of the city in midsummer.

There is something in the salt air of the sea, which has a renovating and life-giving power, to all whose brains have been overtaxed; and to many whose nervous systems have been impaired by intense excitements, whether arising from business anxieties, or domestic calamities. There is also a moral effect for good, in the roar of the ocean, and in the sense of vastness which comes over the mind, as the eye gazes upon it, bottomless, and without a shore beyond; thus causing heart troubles to be swept away in their insignificance.

To persons whose lungs are impaired, or whose throats are in a diseased condition, the air of the sea shore is almost always poisonous, sometimes deadly.

To merchants, clerks, lawyers; to all who follow sedentary occupations, who are kept within four walls for a large portion of every twenty-four hours, no better advice can be given, than to go off among the mountains; climb to their tops; descend into their valleys; penetrate their recesses; on foot, on horse, in every conceivable mode of locomotion; and they should consider every hour of daylight lost, which does not find them in interested motion, in the open air.

The general rule is, to effect a change of air. Any change is more or less beneficial. There is no locality in any dozen miles apart, whose atmosphere has not ingredients differing in some respects from that of other localities, and the human system greedily drinks in those new or strange ingredients, just as one takes in, with unwonted delight and benefit, the food of a table a few miles from his own home. Both mind and body, the world over, yearn for variety, for change. So that a man living for years in the purest atmosphere on earth, will be benefitted by a change to one which, although relatively less pure, has either different ingredients, or the same in different proportions. To all who can, we say, go somewhere, go anywhere, rather than remain at home all the time. Go with as light a heart as possible; go determined to get good, and do good, and you will seldom fail of both. But in going, leave all "airs," and mocks, and pretences, and shams behind. Assume nothing; exact nothing; claim nothing beyond what is spontaneously offered by those, with whom you may come in contact. In all situations, be courteous, and respect yourself, and you will have courtesy and respect shown you. Acting thus, you will return home healthier, happier, wiser and better, than when you went away.

